# Dutch teratological collections and their artistic portrayals

**DOI:** 10.1002/ajmg.c.31902

**Published:** 2021-05-13

**Authors:** Lucas L. Boer, Laurens de Rooy, Roelof‐Jan Oostra

**Affiliations:** ^1^ Department of Imaging Section Anatomy and Museum for Anatomy and Pathology, Radboud University Medical Center Nijmegen The Netherlands; ^2^ Department of Medical Biology Sections Clinical Anatomy & Embryology and Museum Vrolik, Amsterdam University Hospitals ‐ location Academic Medical Center, University of Amsterdam Amsterdam The Netherlands

**Keywords:** art, copperplates engravings, lithographs, malformations, museum, teratology

## Abstract

Several teratologic collections containing specimens with malformations and syndromes are maintained in a number of Dutch anatomical museums. Technically, these are not works of art or antiquities. However, many have been depicted in illustrations of such high quality that they merit discussion here. We review a selection of specimens and their artistic portrayals which find their origin in four Dutch teratological collections. These museum specimens are more than just intriguing objects for the inquisitive museum visitor. As we will substantiate, these specimens—and their artistic depictions—can be used to find and describe rarely occurring birth defects, provide etiopathogenetic information and are a source of novel diagnosis. Additionally, we briefly discuss the ethical aspects and motivations of exhibiting these specimens, as these collections have to be protected meticulously by the new generation of museum professionals, who eventually determine what kind of past our future will have. It is therefore imperative that these collections of antique specimens are treasured as their importance is easily overlooked.

## 1 INTRODUCTION

**FIGURE 1:**
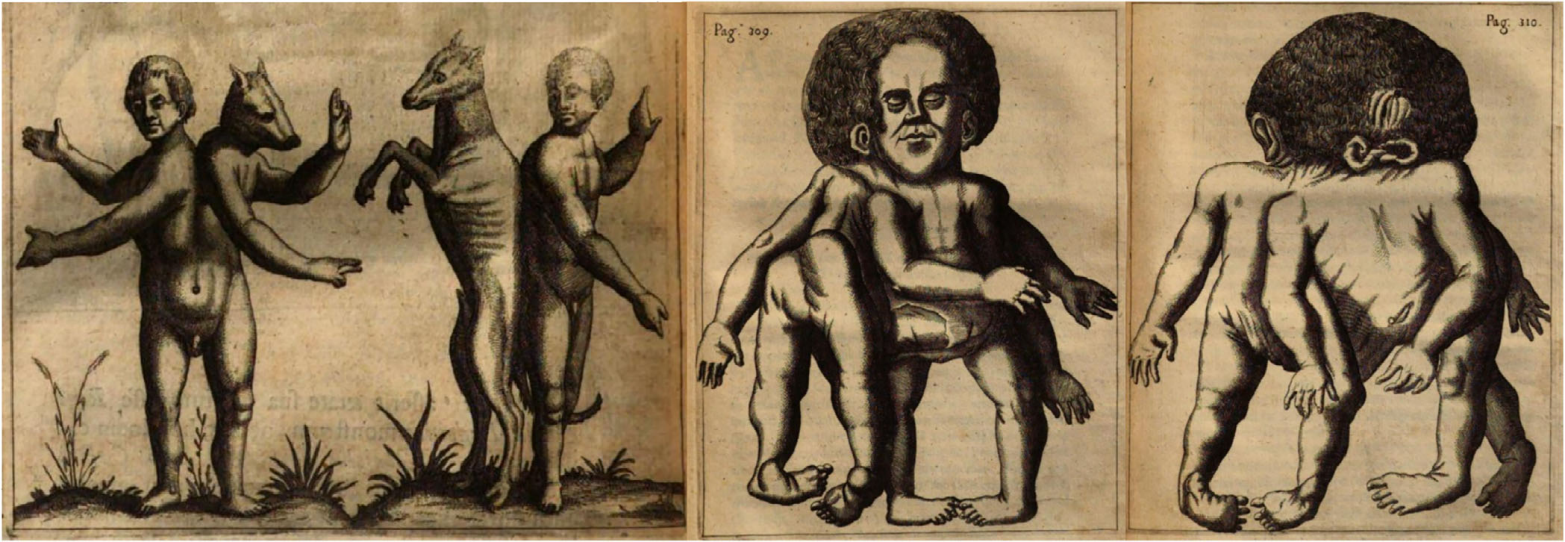
Depictions from Fortunius Licetus (1577–1657) *De monstris* in which both imaginative and relatively naturalistic copperplates of genuine anomalies—in this case a cephalothoracoileopagus conjoined twins—are depicted side by side (Liceti, 1634)

Natural phenomena have always intrigued mankind. The birth of a child with a malformation has been subject of wonder and unbridled fantasy (Bates, 2005). Congenital anomalies tantalized human inquisitive powers (Pachajoa & Rodriguez, 2011). However, these births were more than just cases of rare congenital anomalies, they were initially perceived and considered as omens, hybridizations, divine interventions or even punishments of supernatural origin. Mystification and vagueness were closely intertwined to these births (Beckwith, 2012). It was not until the 16th century that the earliest—although more symbolic—descriptions and depictions of congenital anomalies appeared (Beckwith, 2012). A plethora of quintessential prodigy books flourished in this era. Anomalous births were enigmatically perceived and depicted abundantly (Liceti, 1634). Interestingly, the common characteristic to all available sources of that time is the juxtaposition of imaginative creatures of both human and animal origin aside the depiction of genuine malformations (Figure 1). While superstition and fantastical explanations of congenital anomalies predominate—given the conceptions of procreation and God's role was a wide spread vision—the study of congenital anomalies as a natural philosophical discipline did not occur until the mid‐17th to early 18th century. Curiosity began to replace the superstition through which congenital anomalies were perceived until then (Morin, 1996). Anomalies were beheld empirically: the omens were substituted by meticulous observations and dissections.

During the early decades of the 19th century—seen as the golden age of descriptive and gross teratology in Europe—morphological studies of malformations attained levels of excellence (Bates, 2005). This period resulted in a heritage of teratological preparations and their descriptions, embellished by sophisticated illustrations. Many of these depictions reflected anatomical and artistic skills that are rarely matched today. Exceptional artists were widely available to facilitate the transformation of precise anatomical observation into iconographic elegance. Especially those specimens presenting with dramatic malformations attracted attention (Beckwith, 2012). During this period thousands of teratological specimens were collected throughout Europe. These collections were imperative for the acquisition of historical and clinical (dysmorphological) knowledge, nowadays they can be seen as an (antique) remnant of these times. Although many European museums still house substantial numbers of teratological specimens, they rarely exploit their dormant scientific potentials. When researchers do become interested is these collections, a portal to a hidden value can be seen waiting to be collected, therefore a substantial number of teratological specimens are still on (public) display within Dutch institutions and remain matter for social, educational and scientific programs (Boer, 2019).

Some of these collections are paired with copperplate engravings and lithographic illustrations and comprehensive descriptions. In some cases, in which the actual specimen is lost, the descriptions and depictions are of such high quality and level of detail that a diagnosis could still be made centuries after they were collected (Boer, Radziun, & Oostra, 2017). In fact, many diagnoses could not be made if these depictions were absent—as more often than not—the depicted specimens do not exist anymore. In addition to depicting the actual specimen, some represent earlier stages of the dissection or even the still living individual (Sandifort, 1793a).

In times when additional diagnostic imaging modalities did not exist, the collector was forced to dissect a specimen to obtain information about the internal anomalies. It was apparently part of the academic routine to describe and depict the anatomical observations meticulously (often by a hired artist) as this was the only way to transfer (scientific) knowledge. Some of the initial collectors, including Gerard Sandifort, Frederik Ruysch, and Willem Vrolik, were capable of transforming their observations to iconographic works of art by themselves, indicating that not all collectors were dependent of (paid) draftsmen potentially inducing some sort of self‐interpreted image of the actual specimens' presentation. The purpose of this review is to highlight some of the artistic portrayals of teratological specimens from a number of Dutch anatomical museums, and to reflect broadly on the ethics of collecting.

## 2 *MUSEUM ANATOMICUM* (LEIDEN)

**FIGURE 2:**
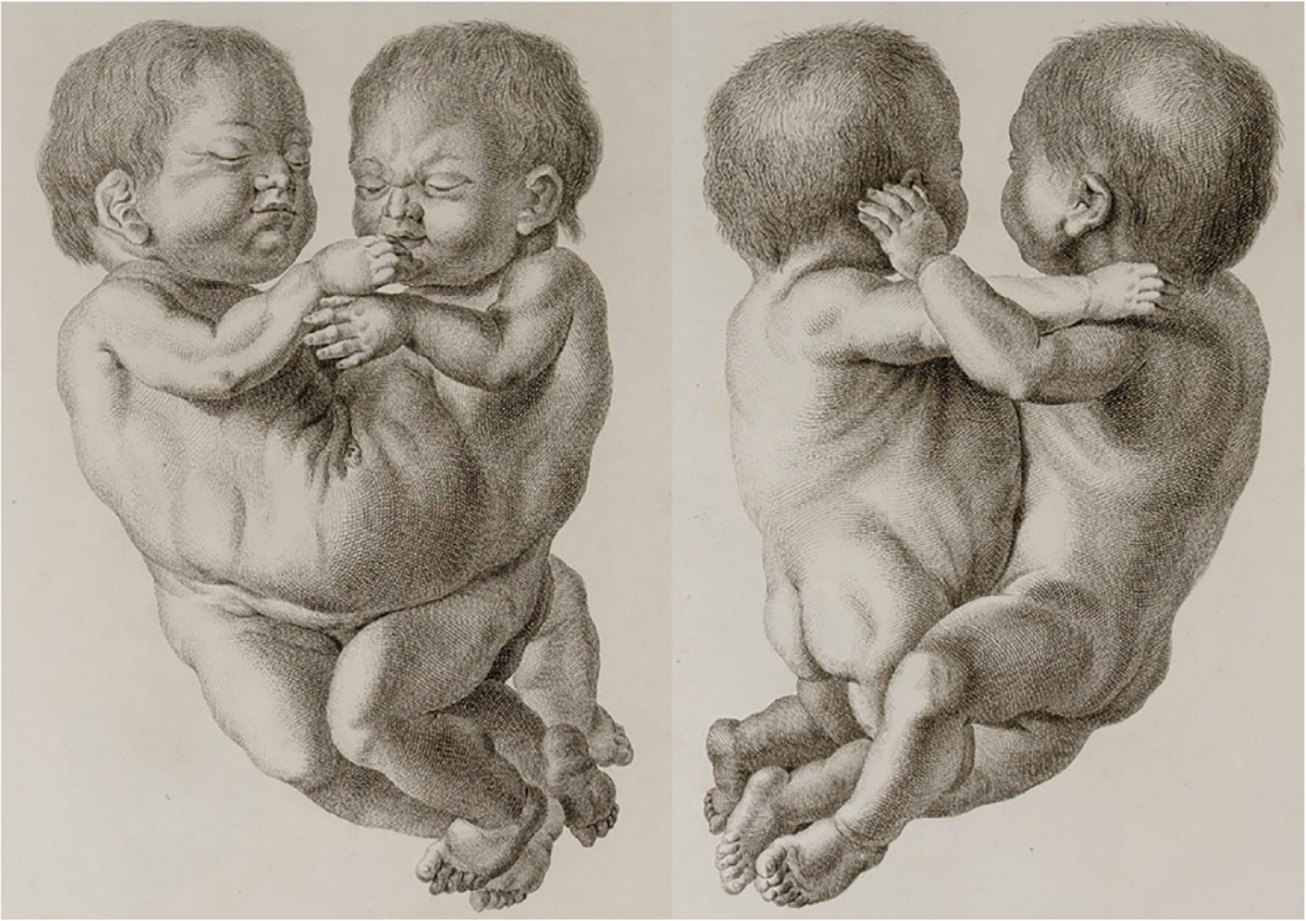
Engraving of an ileoischiopagus conjoined twins from both sides depicted in the second volume of the *Museum anatomicum academiae Lugduno‐Batavae*; showing the elegance and detail of 18th century copperplating by Eduard Sandifort (Sandifort, 1793b)

Founded in 1575, the University of Leiden is the oldest university in The Netherlands. The collections of the Anatomical Museum of the Leiden University Medical Center (historically referred to as *Museum Anatomicum Academiae Lugduno‐Batavae*) currently comprise more than 13,000 specimens including almost 650 teratological specimens (Boer, Boek, van Dam, & Oostra, 2018). This museum houses the oldest Dutch collection of both dried and embalmed anatomical, pathological, embryological, and teratological human specimens (Otterspeer, 2000). Over 350 years, many thousands of specimens were brought together. Because different collectors had their own specific interest, the Leiden museum can be seen as a treasure‐trove for both historical and contemporary (dys)morphological research (Elshout, 1952). Moreover, all collections up to the 19th century were described and depicted in full detail by anatomy professors Eduard Sandifort (1742–1814) and his son Gerard Sandifort (1779–1848). Like his father, Gerard was renowned for his excellent observations and descriptions of both anatomical, pathological, and teratological specimens. This resulted in four volumes including almost 7,500 specimens in which three parts were illustrated abundantly (Sandifort, 1793a; Sandifort, 1793b; Sandifort, 1827; Sandifort, 1835). With this work, which can be seen as one of the pinnacles in teratological illustrations (Figure 2), both father and son became internationally renowned.

### 2.1 Highlights of the Leiden collection

**FIGURE 3:**
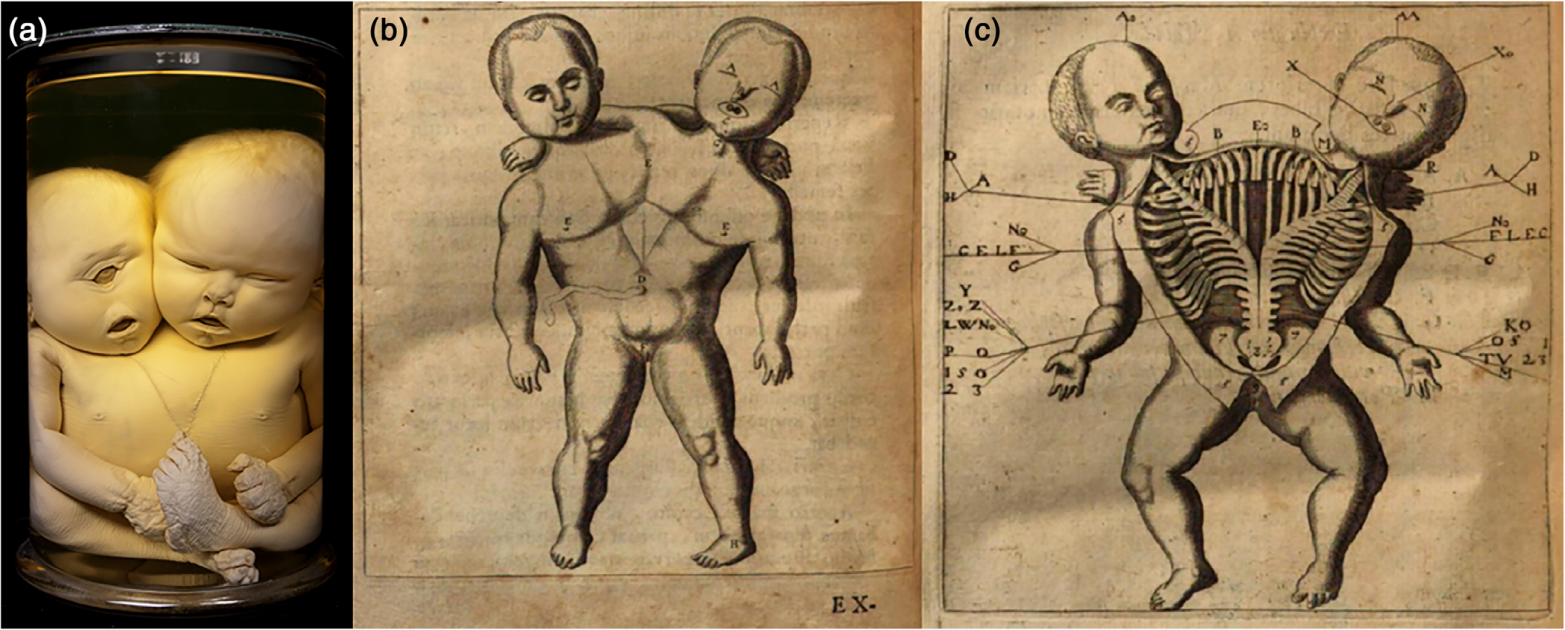
(a) Gross specimen of a parapagus dicephalus discordant for cyclopia from the *Museum Anatomicum* in Leiden. (b, c) Copperplates of another dicephalic twins discordant for cyclopia. Clearly notable is the proboscis and synophthalmia in the right head (Liceti, 1634)

Conjoined twins have always fascinated dysmorphologists throughout time and every anatomical collection houses substantial numbers of (pre)term conjoined twins. In Leiden, 86 cases of conjoined twins or twin‐related anomalies were found in a recent inventory of its teratological collection (Boer et al., 2018). These twins remain a source of contemporary research as etiopathogenetic questions remain hidden behind a multitude of uncertainties (Boer, Schepens‐Franke, & Oostra, 2019). It becomes even more intriguing when one is confronted with conjoined twins in which discordant phenotypes are observable (Boer et al., 2021). One of these very rarely seen combinations concerns a parapagus dicephalus discordant for holoprosencephaly, in this case presenting as cyclopia (Figure 3a), collected by anatomy professor Andreas Bonn (1738–1817). This case was described by both Gerard Sandifort as “*Infans biceps. In capite uno oculi conjuncti sunt*” (Sandifort, 1827) and Willem Vrolik (Vrolik, 1836b). Neither historical illustrations of this specimen were found in the extant collection nor any corresponding treatises. However, and although this phenotype is truly rarely encountered, one of the earliest known reports of a dicephalic conjoined twins discordant for holoprosencephaly (Figure 3b,c) also originates from a Dutch anatomist: Louis (Lodewijk) de Bils (1624–1669). Interestingly, it was only during the re‐inventory and re‐diagnosing project of the Leiden teratology collections—and with that a thorough literature search—that these old engravings were rediscovered. One of the burdens of pre‐ and early modern scientific reports is that they are not systematically indexed and that they are mostly written in Latin, which makes them difficult to access, even to the classically educated. However, Figure 3 illustrates that exceedingly rare anomalies intrigued their original collectors and were already interpreted as being worthy of portrayal.

## 3 THE COLLECTION OF DUTCH ANATOMIST FREDERIK RUYSCH

**FIGURE 4:**
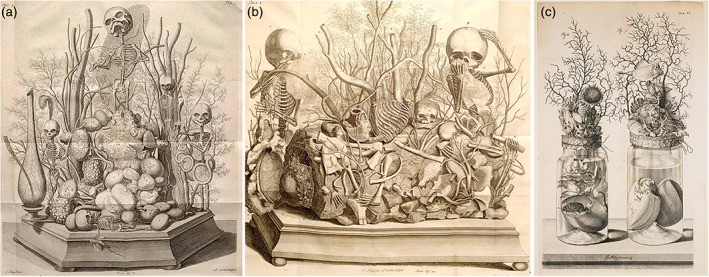
(a, b) Copperplates of Ruysch' dioramas in which the fragility of life is recorded by all sorts of natural elements. (c) Copperplate of the decorated tops of glass vials in which embalmed animal specimens were kept (Ruysch, 1744)

Although the anatomical specimens of Frederik Ruysch (1638–1731) are on permanent display in the Peter the Great Museum of Anthropology and Ethnography (*Kunstkamera*) in Saint Petersburg (Russia), the collection originated in The Netherlands during the late 17th and early 18th centuries (Boer, Radziun, & Oostra, 2017). Ruysch was a Dutch professor in anatomy and botany, who built up a collection of more than 2,000 specimens. Currently, more than 900 are still present in the *Kunstkamera*. Ruysch became famous for his meticulous technique of *postmortem* vascular injections in which even the smallest blood vessels could be visualized and dissected; a groundbreaking technique in the 17th century (Elshout, 1952). Besides these anatomical visualizations, the injection fluid gave his specimens an almost lifelike expression and artistic appearance. The collection that was shipped to Saint Petersburg consisted of twelve subsequently composed cabinets, comprising human, animal and plant specimens which were described by Ruysch in Latin and issued as illustrated catalogues and collectively re‐issued in 1721 (Ruysch, 1721), and posthumously translated in Dutch (Ruysch, 1744). In addition to his scientific contributions, Ruysch is often seen as an artist (Adkins, 2019; Kidd & Modlin, 1999). Among his most admired works are the dioramas, of which Ruysch made a dozen. Tableaus with artistic arrangements assembled from fetal skeletons, dried body parts, gall‐ and kidney‐stones, injected and hardened blood vessels, and all sorts of natural elements. These tableaus referred to allegorical themes such as death and the transiency of life (*Vanitas*): some skeletons were holding one day flies, others were bemoaning their fate by crying into a nose‐rag made of dried and injected brain meninges. In addition, quotations and moral exhortations which emphasized life's transiency and the vanity of earthly riches festooned these tablets. Singing skeletons playing violins were depicted accompanied by the phrase: “Oh fate, oh bitter fate” (Ruysch, 1744) (Figure 4a,b). Besides these dioramas, Ruysch was known for decorating the tops of the jars in which preserved animal specimens were kept (Figure 4c). Although these artistic expressions are somewhat archaic and sinister in a present day rationale, the world famous traveling exhibit “Body Worlds” by *Von Hagens*, showing plastinated bodies in all sort of artificial poses and implicit *Vanitas* messages can be seen as a modern day version of these little cabinets (von Horst, von Hagens, Sora, & Henry, 2019). In essence, not that much has changed throughout four centuries in which anatomy still mesmerizes museum visitors throughout the world, as the human body is and will be part of life for everybody.

### 3.1 Highlights of the Ruysch collection

**FIGURE 5:**
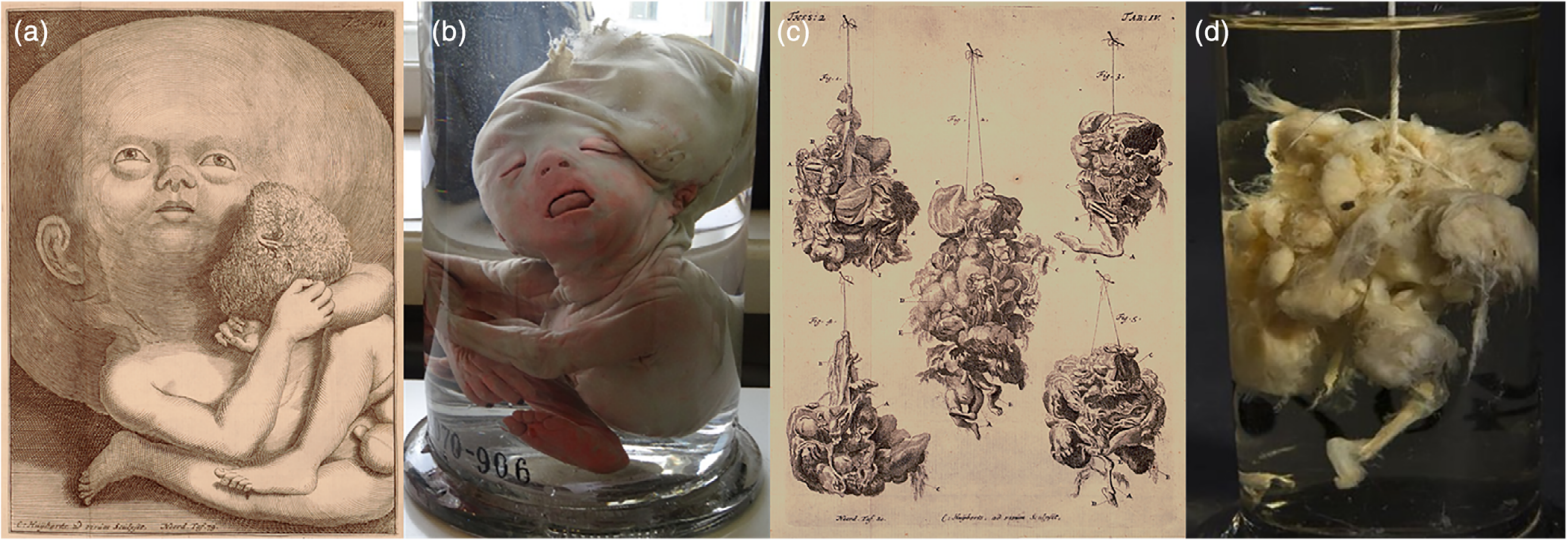
(a) Copperplate of the child which was, according to Ruysch, affected by hydrocephaly. (b) Extant specimens present in the *Kunstkamera* in Russia with an asymmetrical enlarged head and roughly edged opening. (c) Copperplate of the tissue lumps from which limb‐like structures are evident. (d) Extant specimen of one of the tissue lumps later diagnosed to be originated from the cranial vault and interpreted as being an intracranial fetiform teratoma (Ruysch, 1744)

One of the teratological highlights from the Ruysch collection concerns a 6‐months‐old male neonate with a grossly enlarged head, which Ruysch attributed to hydrocephalus. Despite its old age (over 300 years), both the specimens and the engravings are still present (Figure 5a,b). Ruysch' assistance was requested by the midwife who conducted the delivery in which the birth of a child stagnated (Ruysch, 1721, 1744, 1751). Arriving at the scene, both the child and the placenta had already been delivered, however the uterine cavity was still occupied. Subsequently, several shapeless, multi‐structured lumps of tissue were expelled in which Ruysch recognized remnants of no less than 20 miniature extremities without any other recognizable body parts. The size of these extremities suggested a gestational age of 3 months (Figure 5c,d). Inspection of the extant neonatal specimen revealed an asymmetrical sac‐like enlargement of the cranial vault, with a 3 cm wide roughly‐edged opening at the top of the sac. Inspection of the tissue lumps confirmed Ruysch' descriptions in detail: several more or less well‐formed arms and legs were recognizable among various ill‐definable tissue types (Boer, Radziun, & Oostra, 2017). Based on Ruysch' descriptions, the nature of the tissue lumps is not in accordance with the actual gestational age of the child, strongly indicating that they were expelled from the cranial vault during birth (hence the roughly edged opening), a process which has been described by others (Bhattacharya, Cochran, & Loew, 2004; Bolat, Kayaselcuk, Tarim, Kilicdag, & Bal, 2008) and leads to the diagnoses intracranial fetiform teratoma. This case illustrates how important old descriptions and depictions can be in diagnosing conditions in (extant) museal specimens. Undoubtedly, the intracranial teratoma would be very hard to diagnose if the original depictions and story would be absent. It would not come to mind to link the tissue lumps to this specific specimen, which indeed can be diagnosed to be a neonate affected by hydrocephalus at first glance. What makes this case interesting to recall here is the discrepancy between the engraving of the infant and the actual specimen. As said, Ruysch considered this to be a case of hydrocephalus, despite the separate fetiform remnants which he considered to have resulted from multifetation and hence unrelated to the child itself. Apparently, this conviction significantly influenced Cornelius Huyberts, who made the engraving of this specimen and depicted the child with having a spherically enlarged skull, typical for severe hydrocephalus, whereas the specimen itself presents with a much more asymmetric enlargement of the head. Interestingly, this well documented and depicted case report—which is often referred to in literature—has never been diagnosed as such prior to our investigations but instead has always been considered a case of hydrocephalus (Boer, Radziun, & Oostra, 2017).

## 4 *MUSEUM VROLIK* (AMSTERDAM)

**FIGURE 6:**
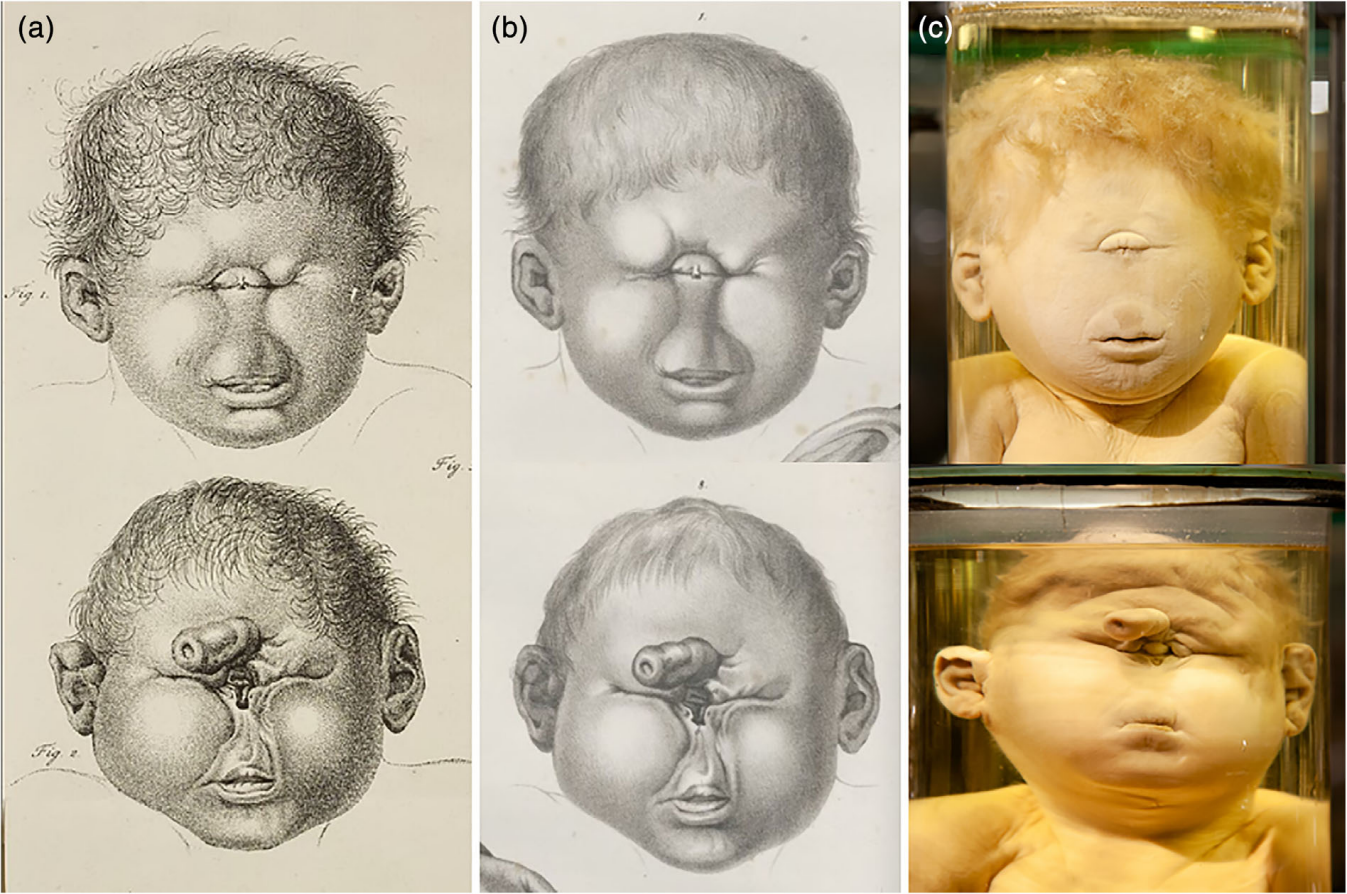
(a) Copperplate engraving by Willem Vrolik (Vrolik, 1836b). (b) Lithograph from the tabulae (Vrolik, 1849) showing copying of earlier depictions. Copperplates were, most likely, made before the skulls of the children were taken out. The lithographs can be seen as a “restyled” version of the copperplates. The exaggerations of fine details, such as around the area of the eye and the philtrum, in both depictions which are not immediately evident in the extant specimens is due to the fact that by removing the skull and creating a wet “taxidermy” specimen fine details and facial profile are lost (c). Moreover, the tooth of time and the use of preservation fluids also have its repercussion on fine details

**FIGURE 7:**
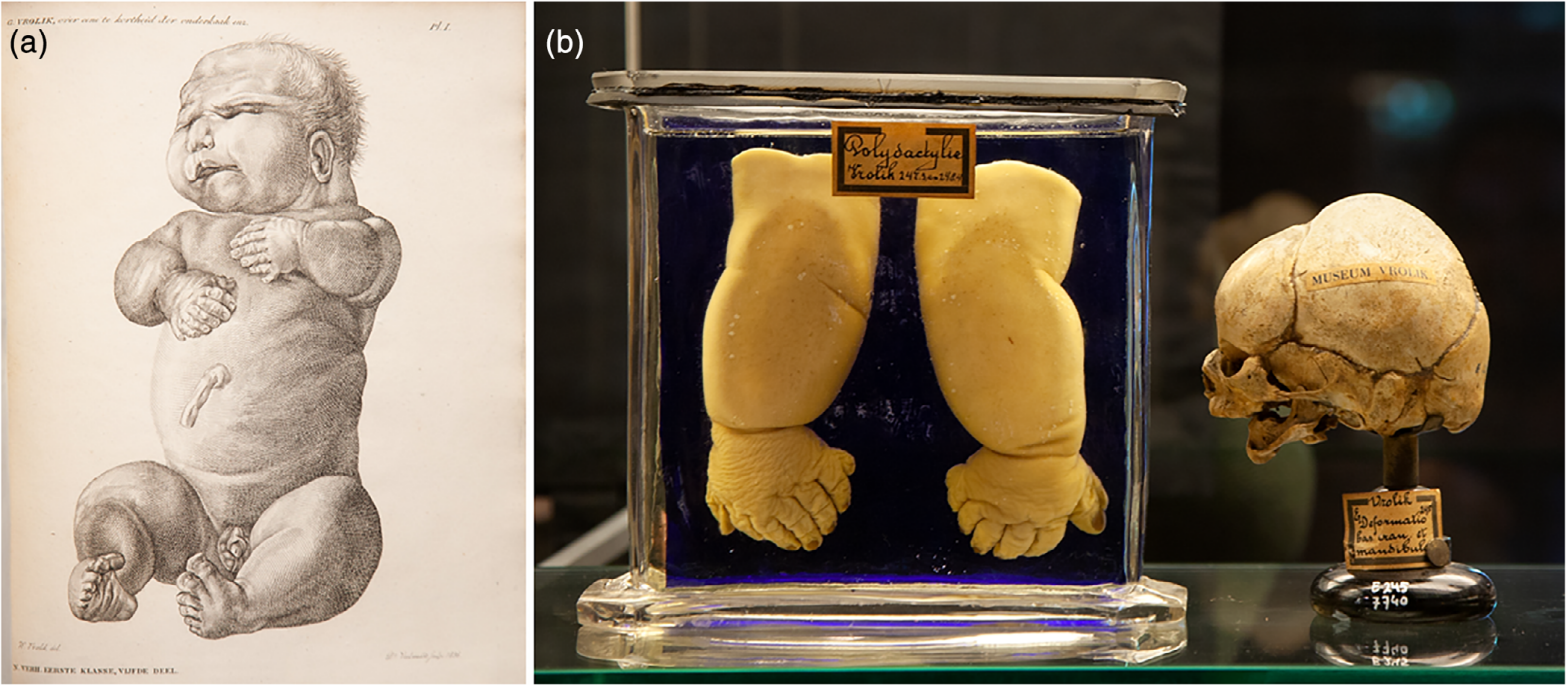
(a) Copperplate of the specimen depicted by Willem Vrolik and described as a condition comprising asymmetry of the skull, severe micrognathia, short limbs, polydactyly of the hands and the feet, and multiple urogenital malformations, including urethral obstruction, micropenis, hypospadia, and cryptorchidism later diagnosed as Majewski syndrome (short rib‐polydactyly type II) (Vrolik, 1836a). (b) Extant preparations of this case in which only the hands and skull were kept as museum specimens after its dissection

**FIGURE 8:**
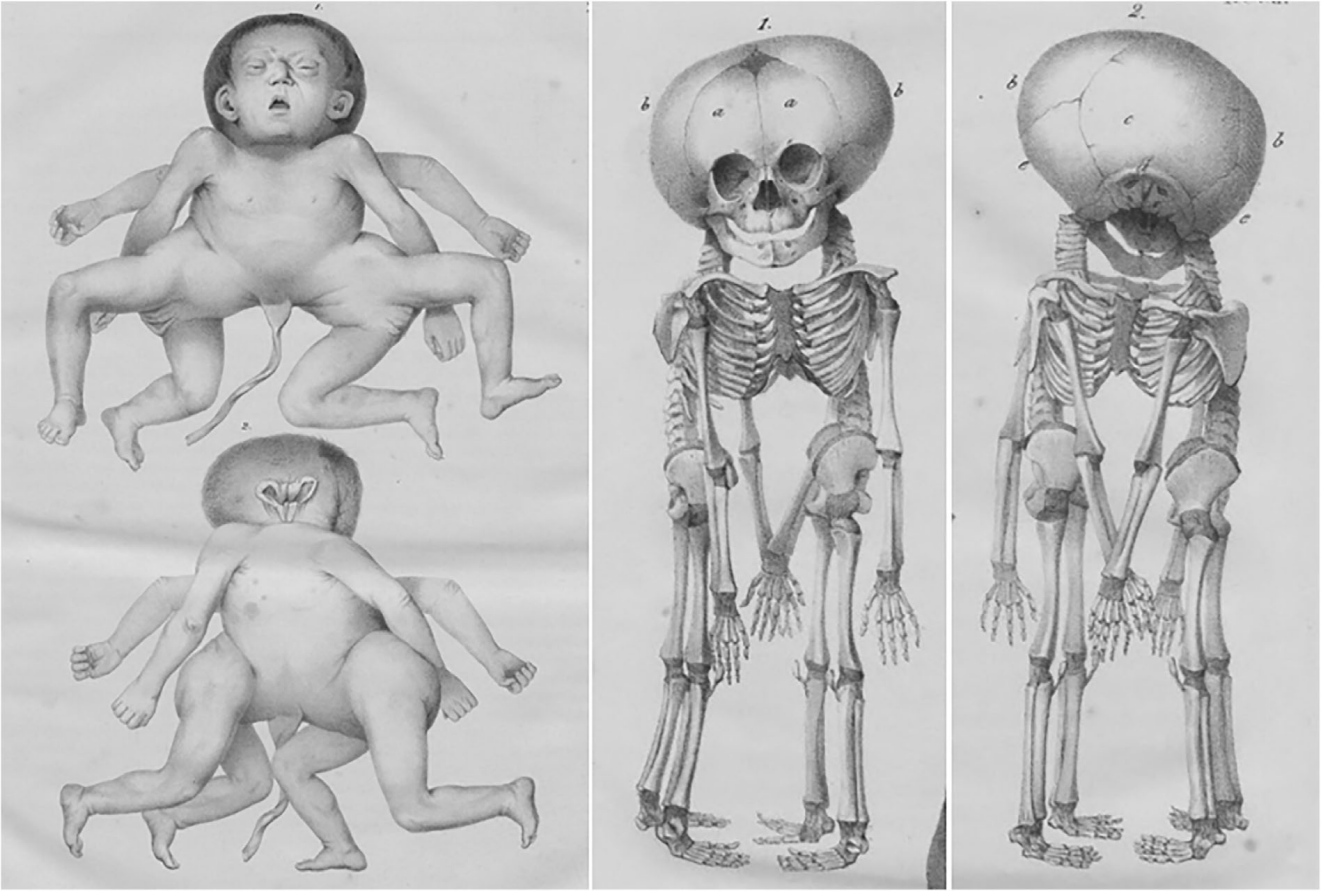
The 96th and 97th plate of the Vrolik *Tabulae* showing a classic example of its lithographs in which both the entire child (in this case a cephalothoracoileopagus twins) and subsequent preparations are depicted. (Vrolik, 1849)

The collections of *Museum Vrolik*, the anatomical museum of the University of Amsterdam, currently consists of around 15,000 preparations and specimens of both human and animal anatomy. Predominantly, the collection was brought together by anatomy professors Gerard Vrolik (1775–1859) and his son Willem Vrolik (1801–1863). In the early 19th century, this collection grew rapidly and it became known as *Museum Vrolikianum* (de Rooy & vd Bogaard, 2009; Vrolik, 1840). *Museum Vrolik* currently maintains approximately 500 teratological specimens which find their origin between 1750 and 1950. More than his father, Willem Vrolik developed a specific interest in congenital anomalies, eventually becoming an internationally renowned teratologist. For his first teratological publication entitled: *Over den aard en oorsprong der cyclopie* (on the nature and origin of cyclopia) (Vrolik, 1836c), Willem made his own drawings (Figure 6a). These specimens currently still reside in *Museum Vrolik* (Figure 6c). In the years before, Willem had become quite a skilled draftsman. He had already illustrated some of his father's publications and most of his own anatomical studies (Vrolik, 1823a; Vrolik, 1823b; Vrolik, 1827a; Vrolik, 1827b). Willem Vrolik made his last contribution as an illustrator of teratological specimens when his father dissected and described a new‐born with multiple gross malformations in 1836 (Vrolik, 1836a) (Figure 7a). This case was later diagnosed as Majewski syndrome (Oostra, Baljet, Dijkstra, & Hennekam, 1998). Although the most relevant parts of the child—the skull, hands and feet—were added to the Vrolik collection after the dissection (Figure 7b), this same dissection destroyed the integrity of the specimen. This was a typical problem for scientific anatomists from the time before imaging techniques. To obtain knowledge about the anatomy of a malformed fetus it had to be dissected. Inherently, it could only be preserved in parts—the “whole” could never be restored accordingly. Old illustrations, showing the entire specimen, are therefore an eminent part of their story. In 1848, Willem completed his most important work in the field of teratology, the *Tabulae ad illustrandam embryogenesin hominis et mammalium tam naturalem quam abnormen* a book consisting of 100 lithographed plates depicting a wide variety of about 250 congenital defects, of which 60% of the cases came from the *Museum Vrolikianum* (Vrolik, 1849) (Figure 8). Both Willem Vroliks drawings of cyclopia (Figure 6b) as well as the illustrations of the case of Majewski syndrome were copied in this book.

### 4.1 Highlights of the *Vrolik* collection

**FIGURE 9:**
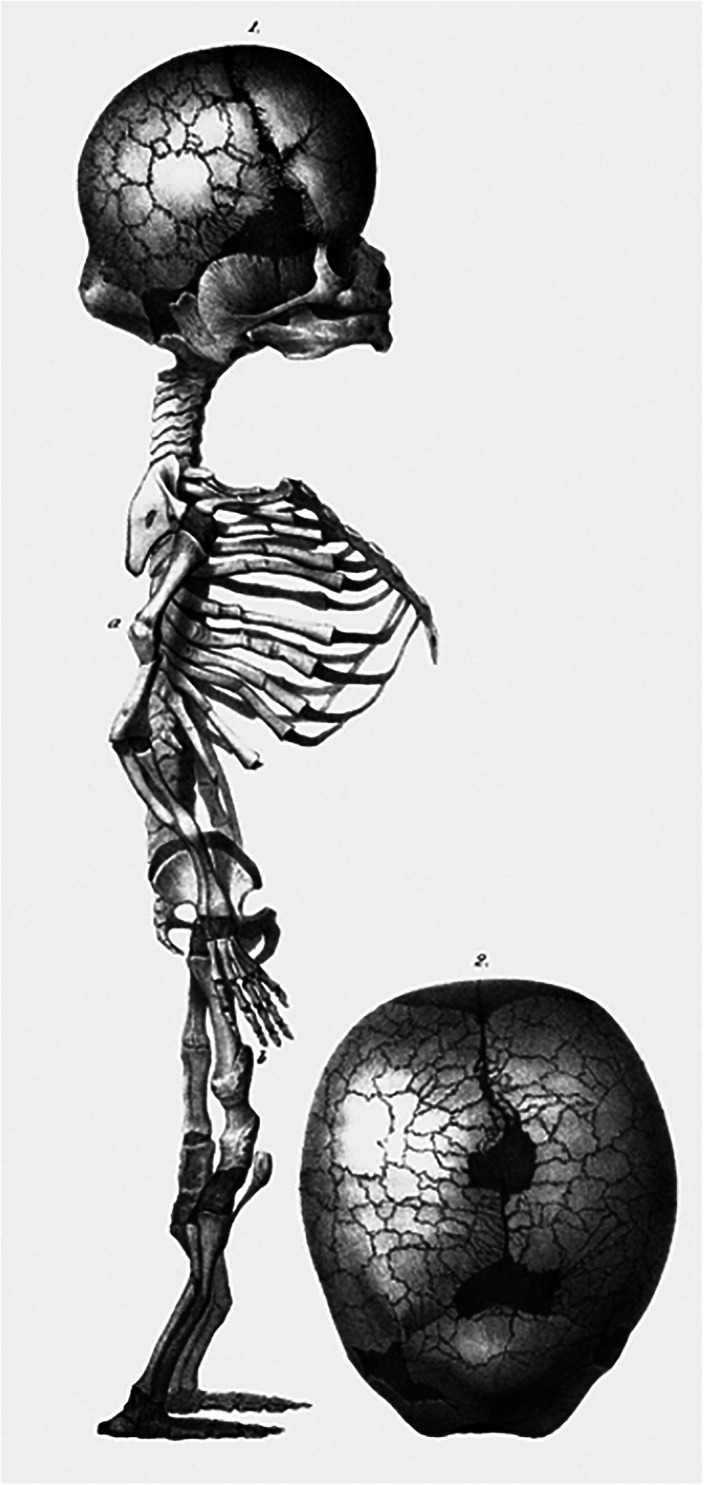
Lithograph of the specimen with “flawed bone formation” later diagnosed as osteogenesis imperfecta type II. From: *Tabulae ad illustrandam embryogenesin hominis et mammalium tam naturalem quam abnormen* by Willem Vrolik (Vrolik, 1849)

The lithographs from the *Tabulae* (Vrolik, 1849) were created by various artists but mostly by Christian Meijer (1806–1875) and Bernard van Loo (1816–1892). As it appears, Vrolik instructed them adequately, since most of these drawings display a detailed accuracy that makes it possible to diagnose the depicted condition even in the absence of the corresponding specimen. For example the case of a newborn that lived for 3 days, presenting with what Vrolik described as “flawed bone formation” and for which he coined the Latin term “*Osteogenesis Imperfecta*”. The skeleton of the child is depicted in the 91st lithograph of the *Tabulae*, together with a detailed drawing of the skull (Figure 9). The quality of the art work, exposing numerous fractures in all tubular bones, some with extensive callus formation, as well as a multitude of Wormian bones in the cranial vault, leave little room for any other diagnosis than osteogenesis imperfecta type II, which to date still bears the eponym Vrolik's disease (Baljet, 2002; Oostra et al., 1998).

**FIGURE 10:**
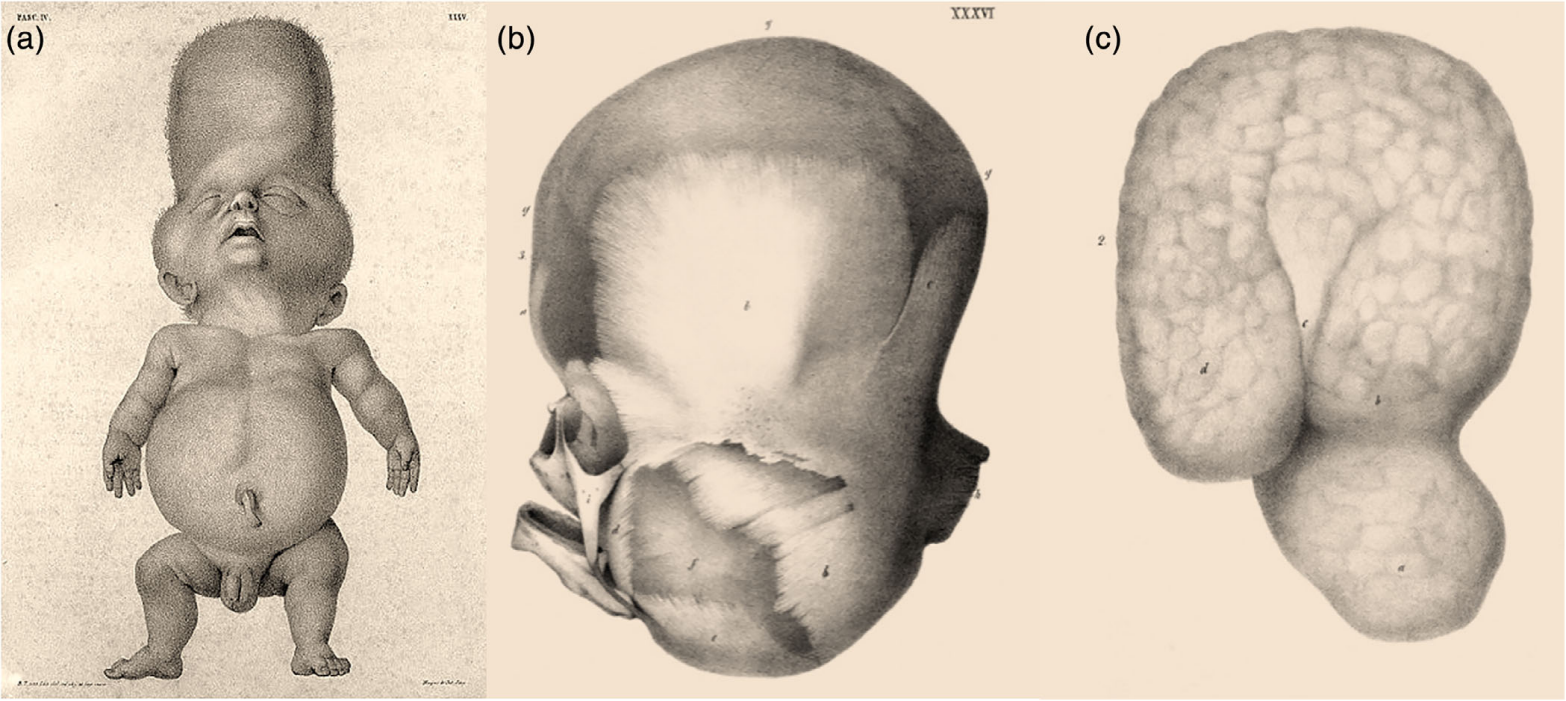
(a) Lithograph of the specimen with hydrocephaly. (b/c) Skull and brain of the same specimen later diagnosed as thanatophoric dysplasia type II. From: *Tabulae ad illustrandam embryogenesin hominis et mammalium tam naturalem quam abnormen* by Willem Vrolik (W. Vrolik, 1849)

Some of the drawings contain valuable additional information pertaining a possible diagnosis, particularly when they depict the specimen prior to (subsequent stages of) dissection or body parts that were never as such admitted to the collection. An example of this concerns the newborn depicted in the 35th and 36th plate of the *Tabulae*, that Vrolik described as presenting with hydrocephalus, an extraordinarily shaped head with bulging of the forehead and cheeks and low‐set ears, short trunk and extremities, and a protruding abdomen. The first of the two plates depicts the infant in its undissected state, which concurs with Vrolik's description (Figure 10a). Additionally, there seems to have been redundant skin folds of the extremities and trident hand shape. The shape of the head is quite reminiscent of cloverleaf malformation, which is confirmed by the drawing of the skull in the second plate (Figure 10b). Taking together with the described disproportioned growth retardation, which is also evident in the extant specimen of the infant's skeleton, the obvious diagnosis is thanatophoric dysplasia type II (Oostra et al., 1998). The second plate also includes a drawing of the extirpated brain (Figure 10c), which was probably not preserved as a separate specimen and is therefore not mentioned in the original catalogue of the collection (Dusseau, 1865). It shows remarkable deformations that reflect the profoundly restricted growth of the skull resulting from the cloverleaf condition. These include enlarged and dystopic temporal lobe, deep transverse temporal fissures and microgyria, which, together with the megalencephaly and callosal hypoplasia that Vrolik encountered when he dissected the brain, have been reported as very common neuropathological changes in thanatophoric dysplasia (Hevner, 2005).

**FIGURE 11:**
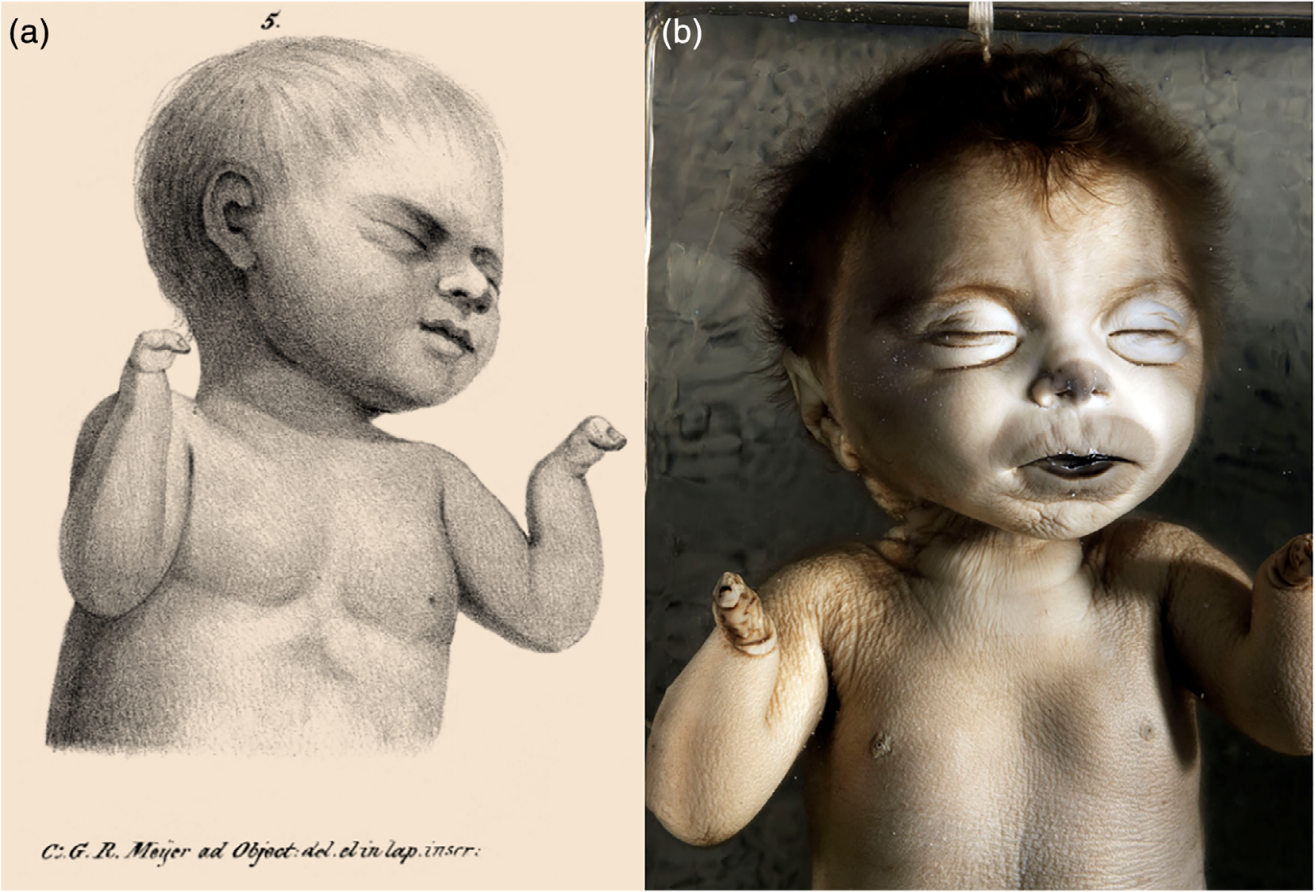
(a) Lithograph of the specimen with monodactyly. From: *Tabulae ad illustrandam embryogenesin hominis et mammalium tam naturalem quam abnormen* by Willem Vrolik (Vrolik, 1849). (b) Photograph of the extant specimen in which the characteristic face of Cornelia de Lange Syndrome is seen

An important realization when analyzing drawings of specimens is that they reflect the proverbial “artist impression” of what is depicted since they are subjected to interpretation, in this case not only by the artist but also—and perhaps even more so—by Willem Vrolik, who most likely will have pointed out which phenotypic aspects of the specimen at hand should or should not be emphasized. A remarkable discrepancy between reality and interpretation is represented by the case of a newborn with monodactyly, described and depicted in the 76th plate of the *Tabulae* (Figure 11a). In addition to the description of the hands being reduced to a single digit, Vrolik explicitly stated that, besides this specific malformation, the child was well‐formed. However, when examining the extant specimen that this description refers to, a much more complex phenotype is encountered, pathognomonic of Cornelia de Lange syndrome (Oostra, Baljet, & Hennekam, 1994). Apparently, the evident microcephaly, generalized hirsutism and distinct facial abnormal traits (Figure 11b), were entirely overlooked by Willem Vrolik and consequently not included in the instructions he gave to the artist. As a result the drawing that was made of the specimen lacks these characteristics completely.

## 5 MUSEUM FOR ANATOMY AND PATHOLOGY (NIJMEGEN)

**FIGURE 12:**
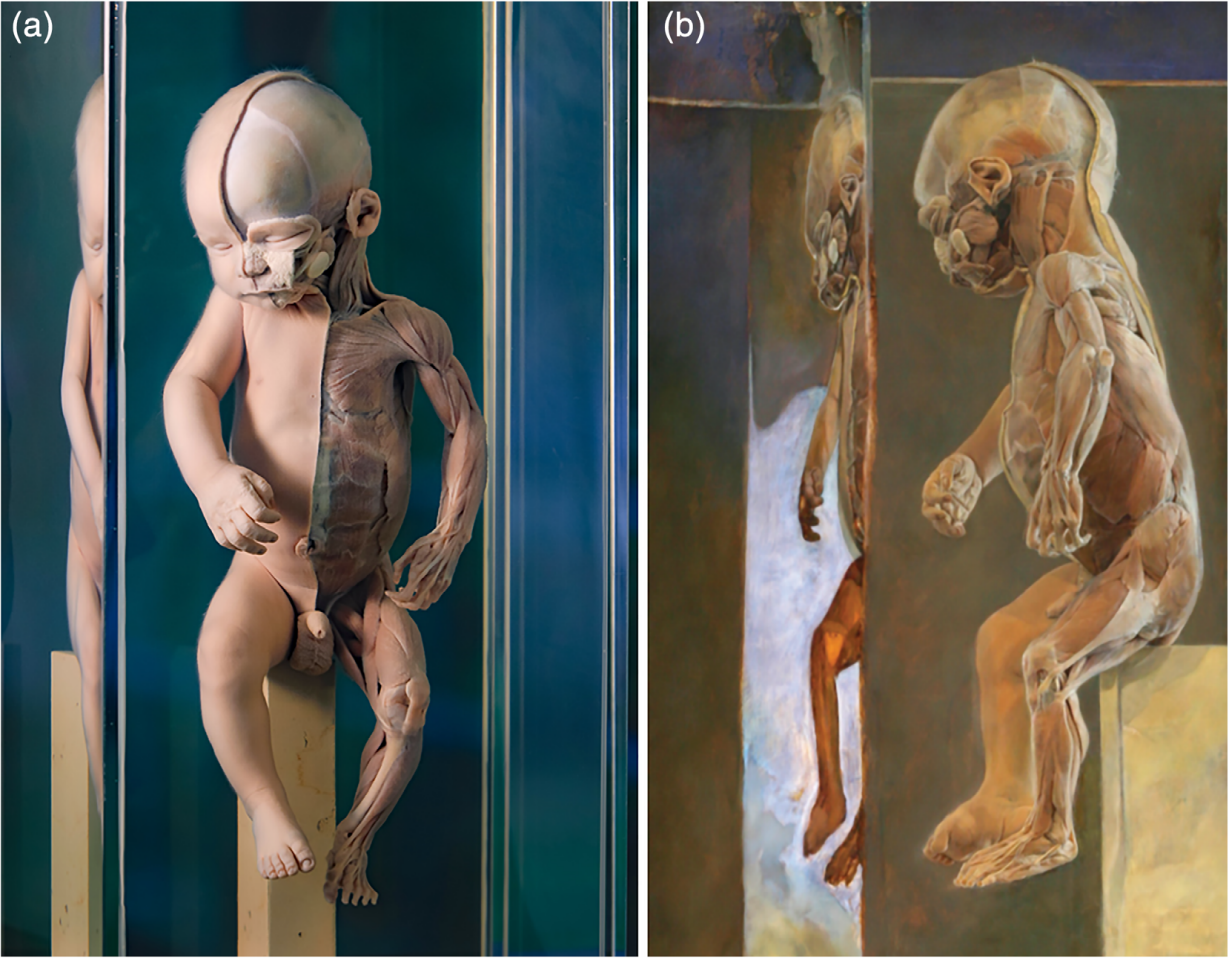
(a) One of the oldest anatomical specimens from the anatomical museum in Nijmegen which was dissected in 1952. The left side of the most superficial muscles of a neonate are dissected. Note the posture with its head almost bowing to the public as a kind of submission or an implicit message of its demise. (b) In addition, anatomical specimens are often matter for contemporary artistic impressions as this neonate was painted by Dutch fine painter Erik van de Beek between 1995 and 2000

The Museum for Anatomy and Pathology in Nijmegen (historically referred to as *Museum Anatomicum*) currently comprises ~1,500 anatomical, embryological and pathological specimens of human origin. Opened in 1967, the anatomical museum in Nijmegen is the youngest in The Netherlands and the only Dutch collection that is still expanding with newly (dissected) specimens. By its relative recent acquisition, the museum inherently has a modest historical value and therefore an open attitude to expand its collections. Despite its small collection in comparison to the other museums described here, its content is renowned for its high quality of anatomically dissected specimens (Figure 12a) which oftentimes serve as inspiration for varies contemporary artists (Figure 12b). The museum houses a publicly accessible teratological collection in which 43 of the in total 74 teratological specimens are on permanent display. Noteworthy is that the teratological collection also grows by a few per year. These are predominantly donations of privately owned specimens but also clinical cases for whom the parents have decided to endue their deceased child to a body donation program. When this occurs parents are fully aware that their stillborn could become a museologic specimen. As a matter of fact this premise could be an altruistic argument for parents to donate their child: “medical society should learn from what has happened to my me and my child.” This exhibition has led to an increase of (post)academic education which was one of the incentives for its realization. This relatively small teratological collection was founded by Dr. Albert Verhofstad (1939–2008) who collected these specimens from various hospitals in and around Nijmegen between 1950 and mid‐1980. Although the procedures by which these specimens were obtained has not been disclosed, it is quite probable that no informed consent was (always) obtained from the parents, who in those days were often not even visually confronted with their child and were told that donation to science was inherent. The museum has recognized that this injustice cannot be turned back and therefore has decided to openly communicate the ethical considerations of that time and the need to display these specimens today.

**FIGURE 13:**
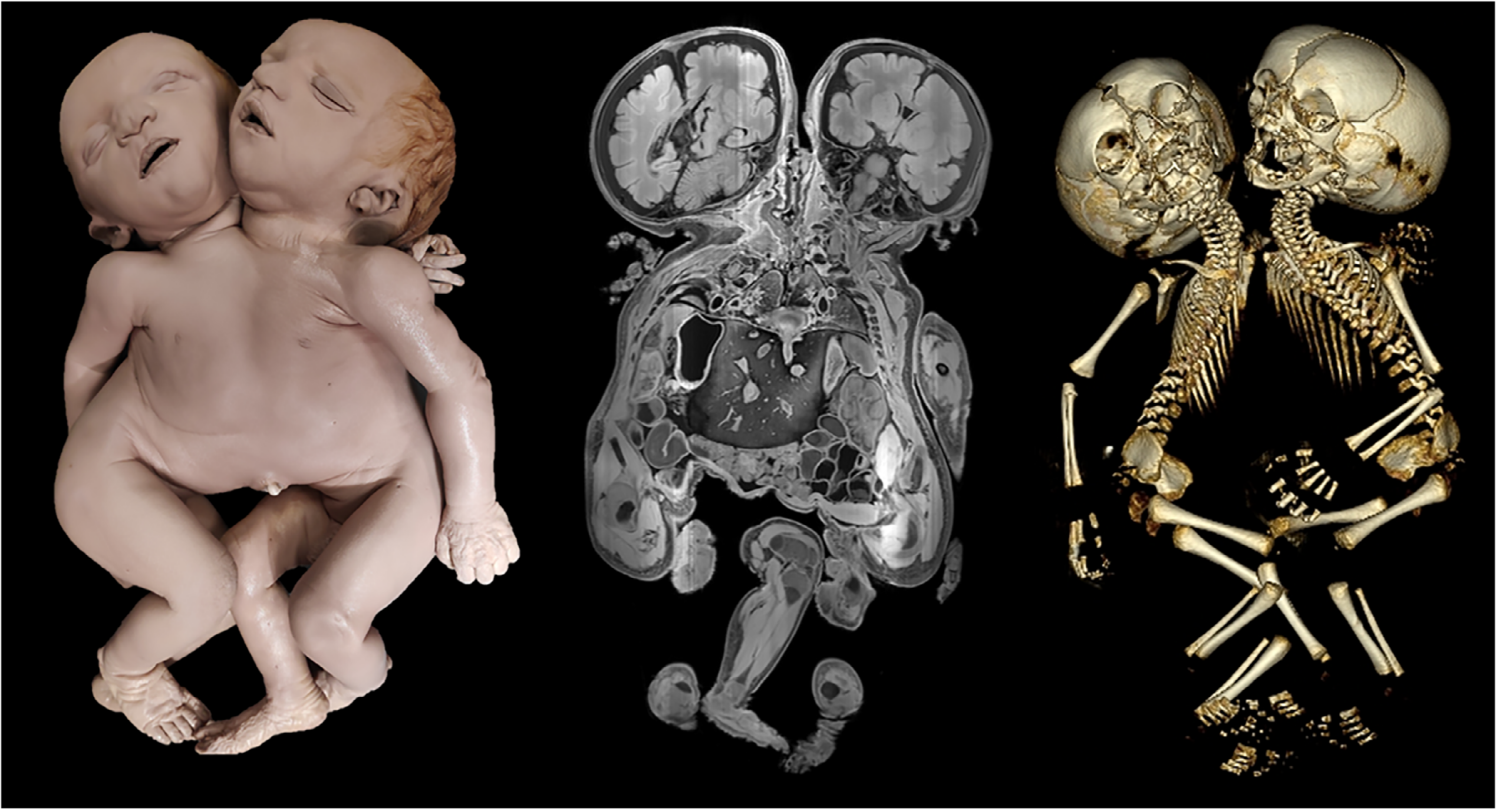
Example of radiological imaging of a conjoined twins from the anatomical museum in Nijmegen in which both magnetic resonance imaging (MRI) and computed tomography (CT) with three‐dimensional reconstructions were used to gain insight into its internal morphological characteristics

Although the Nijmegen collection lacks the historical treatises with their elegant artistic representations, it has a specific value of its own. In contrast to many older specimens, that were necessarily dissected at the time to inspect their internal morphology, the Nijmegen collection predominantly houses fetuses which are completely embalmed and never opened for additional diagnostics. Probably the original collectors considered it advantageous for their exhibitional value to keep the specimens in their native state, thereby making concessions to obtaining a complete diagnostic profile. Whatever their reason was, it puts us in the fortunate position to investigate these specimens with modern imaging techniques. Therefore, besides extensive external examination, each specimen underwent additional radiological imaging (CT and MRI), in order to obtain as much morphological data as possible (without dissection) and to reach a clinical diagnosis, which was considered a prerequisite for public display (Boer et al., 2017) (Figure 13). Moreover, due to the regularly used aggressive preservation fluids, DNA degradation is irrefutable making additional genetic tests mere impracticable. However, every form of additional study—always respecting the integrity of the specimen and its museal/historical value—would be favorable for its story and present beholder. Therefore, additional research should always be considered and explored.

## 6 THE ETHICS OF EXHIBITING TERATOLOGICAL COLLECTIONS

Although many museums exhibit teratological specimens to the general public—as a matter of fact they are often renowned by their vast teratological collections—very rarely do they make explicit their ethical reasoning and considerations regarding the decision to display these specimens. Most specimens were collected during a period in which ethical and moral aspects were approached and acted upon differently in comparison with modern times. In particular with respect to the handling of bodies of dead children and the necessity for consent. The zeal regarding the acquisition of these specimens condoned unsavory methods that dehumanized these specimens and disregarded the desire of their loved ones (DeSesso, 2019). During this era no willed body program was yet available and the “rules,” norms and values which governed the heydays of collecting these specimens was incomparably different from those we know today. The doctor–patient relationship was most probably hierarchical and paternalistic, it did occur neither to doctors to ask parents for permission, nor to the parents to claim that right. The concept of (individual) autonomy and of decisional self‐determination was virtually absent (Nelson‐Marten & Rich, 1999). It deemed sensible to remove the deceased child as soon as possible from the parents' sight. In addition, there were hardly any options for therapeutic interventions, let alone for prevention of recurrence. Most teratological specimens residing in anatomical museums were obtained and anonymized in a way we would now find unacceptable (Jones, Gear, & Galvin, 2003). It is unknown in most (if not in all) cases whether parents consented to the donation of their deceased child. Which information was given to parents regarding their stillborn fetus remains indistinct. Historically rationalized, these deceased children were seen as inert and impersonal. In today's society, however, they are increasingly depicted as personified entities (Morgan, 2002). Although one might find the origin of certain collections unjust within a modern point of view, museums cannot undo this historical injustice which is inherently present in each teratological collections (Gulczynski et al., 2018). According to the International Council of Museums Ethical Code (ICOM, 2013), human remains are invariably considered as “culturally sensitive materials,” implying important ethical evaluations and comprehensive management based on respect in all phases of the management of a museum, from the acquisition, to the conservation and the preparation of the specimens (Monza, Cusella, Ballestriero, & Zanatta, 2019). The nature of these collections is unique and shows the perceptions, attitudes, and superstitions of past epochs (Jones et al., 2003). Even though most museums do not know why, how and when most specimens were collected, it is important to openly communicate certain (general) ethical considerations within the historical context if a museum chooses to exhibit their teratological collections. On the contrary, one could argue that it is unethical not to display these specimens that have been carefully gathered and adapted in the past. They should become more than secretive antique collections only accessible for the happy few clinician and/or researcher. Openly exhibit and communicate its ethical (re)considerations could generate public acceptance and empathy for the norms and values in earlier times. Teratological collections confront viewers with the imperfections of nature, the fragility of human existence and nourish visual tactility. Finally, exhibiting teratological specimens could create a valuable learning experience and potentially contribute to the social acceptance and awareness on developmental defects.

## 7 CONCLUSION

The initial collectors of teratological specimens—and those who described and depicted these peculiar cases—have (unconsciously) determined what we now can investigate. Interestingly, a shift occurred during the last decades in the practice of collecting teratological specimens. In the early days a prerequisite to find anatomical answers was to (completely) dissect the specimens in order to find, describe and depict its inner characteristics. If one wanted to know more about the (dys)morphology of a certain structure, one should dissect ever deeper to the point that the dissected specimen was in such deplorable state that it was no longer suitable to become a museum specimen. And of course perhaps this was the “purpose” of such a specimen: to find and describe its characteristics more so than becoming a specimen to be exposed. Undoubtedly, many specimens perished during this process. In medical history, it is not exceptional that anatomists were outstanding draftsmen at the same time. Just as many famous artists—intrigued by the fabric of the human body—were excellent anatomists. Art and anatomy seem to be in a constant symbiosis—as is underlined by the many artists who visit the anatomical collections as a source of inspiration.

A portal to future knowledge is still present and quietly awaits its awakening. The new generation of museum professionals who research and preserve these specimens will eventually determine what kind of past our future will have and vice versa. It is therefore that these specimens have to be treasured as the importance of such collections of antique specimens is easily overlooked.

## CONFLICT OF INTEREST

The authors declare no conflicts of interest.

## Data Availability

Data sharing is not applicable to this article as no new data were created or analyzed in this study.
